# Editorial: Epigenetic modification in neurological diseases

**DOI:** 10.3389/fgene.2024.1506551

**Published:** 2024-10-29

**Authors:** Mojgan Rastegar

**Affiliations:** Department of Biochemistry and Medical Genetics, Max Rady College of Medicine, Rady Faculty of Health Sciences, University of Manitoba, Winnipeg, MB, Canada

**Keywords:** epigenetics, DNA methylation, regulatory RNA, neurological diseases, traumatic brain injury

Gene regulatory mechanisms control cellular phenotype and function at different levels. Such control mechanisms act at the transcriptional and post-transcriptional levels prior to the translation of the mature transcripts into functional proteins. During these multi-step processes, epigenetic mechanisms play key roles in gene regulation by fine-tuning the active-inactive states of any given gene, while determining their level of transcription. Although, there are many different levels of epigenetic mechanisms, perhaps modification of DNA, RNA, and proteins (i.e., histones) are the best-studied examples of epigenetic mechanisms.

Epigenetic modifications happen in a dynamic and continuous fashion during life. These include molecular modifications of DNA, RNA, and proteins (histones) during cellular renewal and differentiation, and organ formation throughout development and life. It is not surprising that deregulation of such fundamental mechanisms could lead to cellular abnormalities and organ disfunction, causing human disease. While all body parts and organs have their own regulatory processes, the mammalian brain and central nervous system appear to be the most complex part of the body. Accordingly, neurons that are the nerve cells in the central and peripheral nervous system have distinct characteristics that makes them especially important. Of note, change in epigenetic modifications in the central or peripheral nervous system may lead to neurological disorders. This Research Topic on “*Epigenetic modification in neurological diseases*” includes four peer-reviewed articles published in Frontiers in Neuroscience.

Focusing on the role of non-coding regulatory RNA molecules in Alzheimer’s disease (AD), Canoy et al. discuss recent advances in AD pathology. In this systemic review, the authors cover a range of topics on long non-coding RNA (lncRNA) molecules, microRNAs (miRNA), circular RNAs (circRNA), as well as piwi-interacting RNAs (piRNA). In addition to the role of these regulatory RNAs in AD pathology, their potential application as diagnostic markers as well as possible therapeutic targets are also discussed. In this regard, the authors cover implication of these regulator RNAs in different cellular processes including cell propagation and cell death, apoptosis, autophagy, tau phosphorylation, amyloid-beta aggregation, oxidative stress, and neuroinflammation. The authors further explore the workflow of their approach in screening literature and databases that is clearly demonstrated in the first Figure of this article. Additional illustrations are used to present early onset *versus* late onset, and not-defined cases of Alzheimer’s disease along with extra information within the three Figures and one Table of this comprehensive review (Canoy et al.).

The article by Tian et al., presents an interesting concept on the impact of elevated hemoglobin A1c (HbA1c) with increased risk for impaired cognition in a sex-dependent manner. While the link between epigenetic modifications such as DNA methylation with that of HbA1c in type 1 diabetes has been reported (Chen et al., 2020), this study highlights the potential effects of higher levels of HbA1c in cognitive impairment. The authors explain how they have analyzed independent datasets from UK Biobank cohort with and without neuroimaging information, as the gene-outcome and gene-exposure groups, respectively. The authors describe certain types of analysis to include HbA1c levels, brain age gap, and fractional anisotropy. The detailed Figure 1 of this article captures the systemic analysis and study design of the authors and is complemented with two Tables and additional Figures to represent the results (Tian et al.).

A systemic review by Yuan et al., provides engaging contents on the link between epigenetics and depression. This study benefits from the Web of Science core dataset that covers depression epigenetics published studies between 01/2002-to-06/2023. The authors used certain key words in their literature search, highlighting that the link between epigenetics and adolescent depression requires further investigations. The authors discuss the nature of depression, their approach and strategy in dataset screen and analysis, and present their results. Multiple Figures and Tables are included in this article to clearly demonstrate the study design and findings (Yuan et al.).

The perspective article by Smolen et al. focuses on the association between traumatic brain injury (TBI) with a risk to experience neurodegenerative disorders based on cellular epigenetic alterations. The authors make a case by evidence from epidemiological studies to support such association, while acknowledging the need for molecular investigations. The authors explain how certain neurodegenerative diseases such as AD and Parkinson’s disease (PD) may have epigenetic components. In the other hand the authors explain that individuals suffering from TBI may show short-term or long-term change of epigenetic mechanisms in the brain cells that could potentially be a link to the risk of experiencing AD, PD, and/or dementia. In this article, the authors discuss epigenetic modifications such as DNA methylation and histone post-translational modification(s) like acetylation. The authors further discuss the link between TBI pathology and epigenetic modifications, presenting their hypothesis that these could potentially be considered a risk for neurodegenerative disorders. They also provide insightful opinion on how certain interventions could prevent the development of neurodegenerative disorders. Their discussions of such therapeutic intermediation include the use of methyl donors, inhibition of chronic neuroinflammation, as well as the application of inhibitors for histone acetylation or methylation. This article is complemented with a Table that covers a list of genes that are affected by TBI, suggested short-term or long-term change in their expression along with link with the pathology of neurodegenerative disorders (Smolen et al.) This article published in Frontiers in Neuroscience is an interesting read.
